# Microsurgical Reconstruction of Extensive Lower Limb Defects: Latissimus Dorsi Free Flap for Circumferential Soft Tissue Loss Following High-Energy Trauma

**DOI:** 10.3390/jcm14134424

**Published:** 2025-06-21

**Authors:** Edoardo Filigheddu, Federico Ziani, Giovanni Arrica, Sofia De Riso, Anna Manconi, Corrado Rubino, Emilio Trignano

**Affiliations:** 1 Plastic Surgery Unit, University Hospital Trust of Sassari, 07100 Sassari, Italy; edoardo.filigheddu@aouss.it (E.F.); giovanni.arrica@aouss.it (G.A.); s.deriso1@studenti.uniss.it (S.D.R.); anna.manconi@aouss.it (A.M.); corubino@uniss.it (C.R.); etrignano@uniss.it (E.T.); 2 Department of Medicine, Surgery and Pharmacy, University of Sassari, 07100 Sassari, Italy

**Keywords:** lower limb reconstruction, latissimus dorsi flap, microsurgical reconstruction, free flap

## Abstract

**Background/Objectives:** High-energy trauma to the lower limb often results in extensive soft tissue loss with exposure of critical structures, posing a serious threat to limb viability. Early and effective coverage is crucial to prevent infection, promote bone healing, and preserve function. This report presents the use of a latissimus dorsi free flap for circumferential soft tissue reconstruction following a severe crush injury. **Methods:** We describe the case of a young female patient who sustained a high-energy crush trauma with a comminuted, displaced fracture of the middle and distal third of the tibia and complete circumferential soft tissue loss. Due to the extent and location of the defect, a latissimus dorsi free flap was selected for reconstruction. The surgical technique, microsurgical anastomosis, postoperative care, and rehabilitation protocol are detailed. **Results:** The latissimus dorsi flap provided reliable coverage of the entire defect, protected the underlying bone and hardware, and promoted wound healing. No major complications were observed. Functional recovery was satisfactory, with progressive weight-bearing and joint mobility achieved during follow-up. **Conclusions:** In complex lower limb injuries with extensive soft tissue damage, free flap transfer remains a key strategy for limb salvage. The latissimus dorsi flap, due to its size, reliability, and versatility, represents a valuable option for circumferential coverage and restoration of limb function following high-energy trauma.

## 1. Introduction

Lower limb reconstruction using free flaps is one of the corner stones of plastic surgery [1]. In patients with extensive soft tissue deficits, microvascular free flap reconstruction is the optimal strategy for limb preservation [2].

Lower extremity injuries, predominantly resulting from high-energy trauma, are becoming increasingly prevalent due to the continued expansion of industrialization and motorized transportation. Hemmann et al. [3] reported a 4.5% rise in lower extremity fractures in Germany, from 305,764 cases in 2002 to 319,422 in 2017, with hip and femur fractures increasing by 23.5% over the same period. Complementing these findings, Zhang et al. [4] documented 8613 lower limb fractures over seven years at a UK level I trauma center, corresponding to an annual incidence of 215.9 per 100,000 individuals. Notably, 18.6% of femoral shaft fractures and over 40% of tibial shaft fractures were open injuries, reflecting a high incidence of severe soft tissue loss that frequently requires microsurgical free flap reconstruction. These injuries are often characterized by extensive zones of tissue damage extending beyond the primary wound, and the associated inflammatory response can further impair vascular integrity, complicating timely and effective soft tissue coverage [5]. When recipient vessels are compromised, the radial forearm flap can serve as a vascular bridge in vessel-depleted regions [6].

These injuries pose substantial challenges for reconstructive surgeons, as they frequently involve the exposure of critical anatomical structures such as bones, tendons, and joints, necessitating vascularized tissue coverage to prevent complications and facilitate healing. The fundamental objectives in lower-extremity reconstruction include the restoration of adequate wound coverage, the prevention of infection, and the optimization of functional outcomes to ensure limb preservation and rehabilitation [7,8,9].

Over the past decades, the surgical management of complex soft tissue defects has evolved substantially, particularly with the advancement of microvascular free tissue transfer. These techniques have significantly reduced amputation rates and improved long-term outcomes by providing reliable, definitive coverage [10]. Recent systematic reviews have confirmed the high adaptability of microvascular free flaps, with variable failure rates and reoperation needs depending on defect location, patient comorbidities, and flap type, emphasizing their versatility in complex reconstructions [11].

While local flaps or skin grafts may be suitable for minor defects, the limited availability of expendable tissue in the lower extremity often necessitates microvascular reconstruction. Free flaps offer tailored solutions for defect coverage, ensuring high success rates in cases of trauma, infection, or oncologic resections [12]. In crush injuries, where extensive damage affects both osseous and soft tissue structures, reconstructive challenges are further amplified. The pathophysiology involves ischemia, necrosis, and substantial tissue loss due to sustained compression. Optimal management requires a multidisciplinary approach integrating orthopedic and plastic surgery expertise [8]. Multistage protocols, including negative pressure wound therapy (NPWT) for wound bed preparation and early osseous stabilization prior to definitive free flap coverage, have been shown to enhance reconstructive outcomes [13].

## 2. Case Presentation

**Figure 1:**
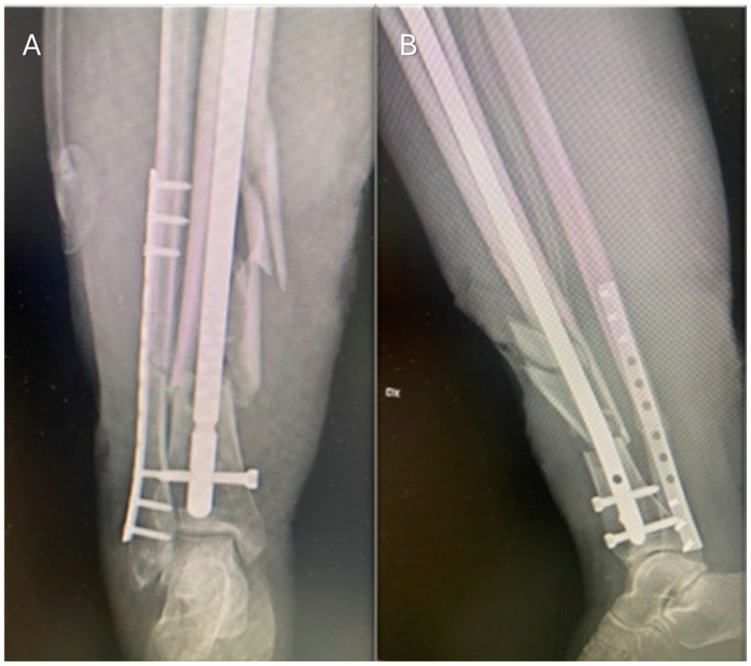
**Post-operative X-rays.** (**A**) Anteroposterior view. (**B**) Lateral view. Fixation of tibia with intramedullary nailing with proximal and distal locking. Stabilization of fibula using plate and screw fixation system.

A 22-year-old woman sustained a crush injury to her right leg in a car accident while studying abroad. The trauma resulted in a closed, multi-fragmentary displaced fracture of the middle and distal thirds of the tibia and fibula, without immediate soft tissue loss. The patient underwent emergent internal fixation with a dynamic intramedullary nail for the tibial fracture and plate/screw fixation for the fibular fracture (Figure 1).

The patient was a healthy non-smoker without comorbidities or prior surgeries.

**Figure 2:**
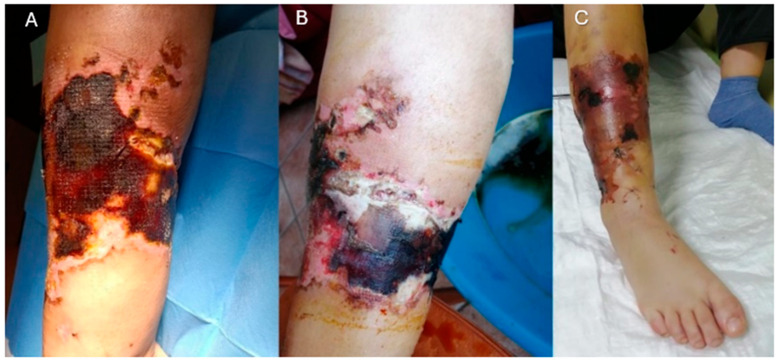
**Preoperative photographs.** (**A**) Medial aspect. (**B**) Posterolateral aspect. (**C**) Anterolateral aspect. Clinical photographs showing progression of soft tissue necrosis and extensive ischemic areas in distal leg following internal fixation of tibio-fibular fracture.

During the early postoperative period, upon returning to her home country, she presented to the local orthopedic unit with progressive edema, ischemic soft tissue compromise, and extensive necrosis, ultimately leading to tibial bone exposure and circumferential soft tissue loss in the middle and distal thirds of the leg (Figure 2). This was attributed to post-traumatic and post-surgical ischemic injury secondary to muscle compression. At clinical presentation, a thorough neurological examination was performed. Motor function and sensory response in the affected limb were fully preserved, with no evidence of foot drop or peripheral nerve deficits. The patient maintained active dorsiflexion and plantarflexion, and no areas of hypoesthesia or allodynia were detected.

**Figure 3:**
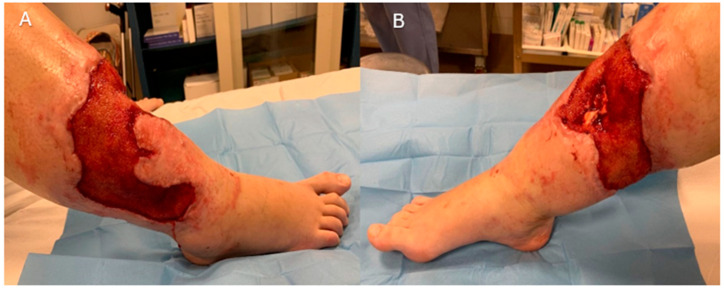
**Post-debridement and application of NPWT.** (**A**) Lateral aspect. (**B**) Medial aspect. Extensive soft tissue loss visible on both medial and lateral aspects of distal leg, with bone exposure and with healthy, well-perfused wound bed prepared for further reconstructive management.

A multidisciplinary management approach was initiated. Under the supervision of the Plastic Surgery Unit, the patient underwent serial debridement and wound care, leaving a 20 × 25 cm circumferential area (500 cm^2^). Negative pressure wound therapy (NPWT) was applied for 10 days to optimize wound healing, promote granulation tissue formation, and minimize the risk of soft tissue and bone infection. Once a well-vascularized wound bed with no bacterial contamination was achieved, the patient was referred for definitive reconstruction (Figure 3).

Preoperative angiography confirmed the patency of major vascular structures, ruling out contraindications to microsurgical reconstruction. Given the extensive tissue loss and the need for robust bone coverage, local flap options were deemed unsuitable, and a latissimus dorsi free flap was planned.

Upon admission, she was already on oral antibiotic therapy—amoxicillin-clavulanic acid 875 + 125 mg—due to soft tissue inflammation at the fracture site; this regimen was maintained through reconstruction and discontinued 48 h postoperatively, once inflammatory markers normalized.

**Figure 4:**
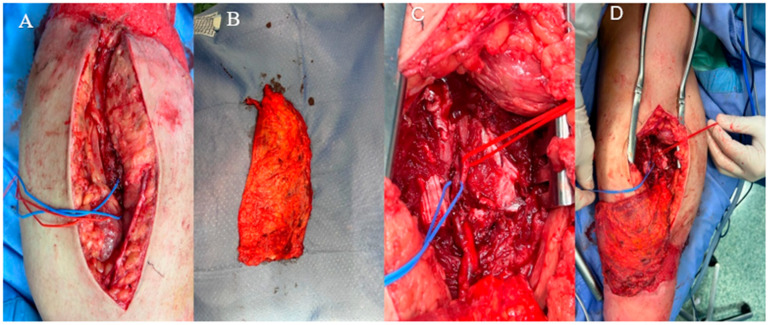
**Intraoperative photographs.** (**A**) Dissection between the medial head of the gastrocnemius and the soleus muscle to expose the posterior tibial artery and vein; (**B**) harvested latissimus dorsi flap with its vascular pedicle; (**C**) end-to-end microvascular anastomosis between the thoracodorsal artery and the posterior tibial artery; (**D**) inset of the muscle flap into the defect site.

**Figure 5:**
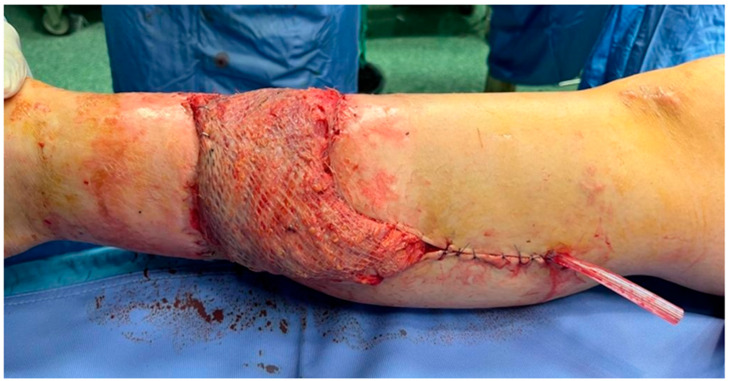
**Intraoperative photograph.** View of the latissimus dorsi free flap inset, covered with a meshed split-thickness skin graft and a surgical drain.

A latissimus dorsi free flap was harvested, and its vascular pedicle was anastomosed end-to-end to the posterior tibial artery and vein through a medial approach (Figure 4). A split-thickness skin graft was applied over the muscle flap for additional coverage (Figure 5). The total surgery time was 180 min and flap ischemia was 60 min. A below-knee plaster cast was used for 25 days to avoid any movement of the foot and to permit postoperative edema without increasing compartmental pressure. The post-operative period was uneventful, and hospitalization lasted 13 days; during this time, the patient underwent serial neurovascular assessments during the first 48 h to monitor for compartment syndrome, all of which remained normal. Anticoagulation prophylaxis with enoxaparin 4000 IU/die was administered during hospitalization and continued for 30 days post-discharge. Analgesic management included IV paracetamol and NSAIDs, without opioids. The limb was elevated with pillows and repositioned regularly to minimize edema. In the early postoperative phase, controlled limb lowering (‘dangling’) protocols are recommended to promote vascular adaptation and reduce the risk of venous congestion in lower extremity free flaps [14]. Primary wound healing was achieved without anastomotic complications, local infection, or recurrent bone exposure. The patient subsequently underwent a progressive weight-bearing rehabilitation program with assisted ambulation for an additional 20 days.

Follow-up visits for medications were fixed at 9, 12, 15, 18, and 21 days after surgery.

At 2 weeks, the flap survived successfully, and stitches were removed; the donor-site was healed, with a 15 cm linear vertical scar on the left side of the back, normochromic and normotrophic, with a regular evolutionary course and without signs of infection or dehiscence. Donor-site morbidity remains a significant consideration in reconstructive surgery, with recent data highlighting its impact on postoperative pain, function, and quality of life [15].

**Figure 6:**
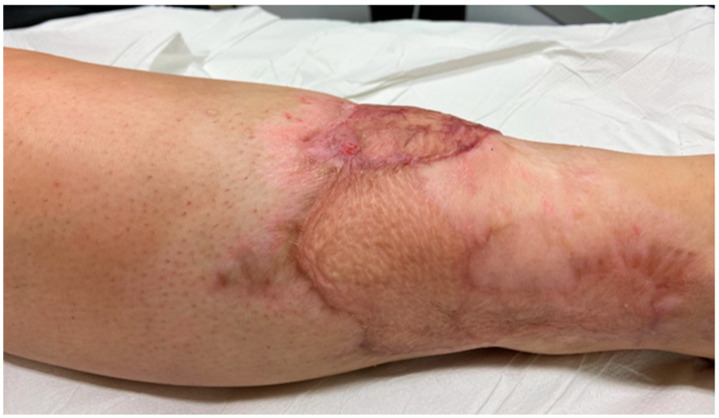
**Postoperative photographs.** Six-month follow-up showing adequate wound healing and satisfactory esthetic outcome, with good integration of flap and skin graft.

At the six-month follow-up, the flap exhibited mild atrophy with favorable contouring, satisfactory esthetic integration, and adequate scar maturation, including areas covered with the skin graft (Figure 6). Radiographic assessment confirmed complete bone healing. The patient achieved full functional recovery, resuming normal ambulation and daily activities with an overall successful reconstructive and esthetic outcome.

The long-term functional outcome was assessed at 18 months using the Lower Extremity Functional Scale (LEFS), demonstrating excellent results with a total score of 76/80, corresponding to 95% of maximal functional capacity [16].

## 3. Discussion

Extensive lower extremity defects following high-energy trauma represent a significant reconstructive challenge, and the choice of free flap type is central to achieving optimal functional and esthetic outcomes. Historically, muscle free flaps (such as latissimus dorsi, rectus abdominis, or gracilis flaps) have been favored for their robust vascularity, ability to obliterate dead space, and potential to reduce infection risk in contaminated wounds. Experimental and early clinical data supported the notion that muscle flaps deliver a generous blood supply to the wound bed, thereby enhancing bacterial clearance and promoting bone healing in complex injuries [17,18].

In contrast, fasciocutaneous free flaps—particularly the anterolateral thigh (ALT) flap—have gained popularity in recent years due to their versatility and minimal donor-site morbidity. Furthermore, the use of ALT flaps with fascia lata extension has been successfully employed in complex post-traumatic reconstructions involving both soft tissue coverage and ligament repair [19]. These flaps offer several distinct advantages: their thin, pliable nature allows for improved contouring and esthetic integration, and they can be re-elevated more easily for secondary orthopedic procedures or revisions when compared to bulky muscle flaps [20]. Moreover, retrospective series have indicated that patients reconstructed with fasciocutaneous flaps may experience a faster return to weight-bearing, although they are sometimes more likely to undergo elective debulking procedures to optimize cosmetic outcomes [17,21].

Despite these differences, several comparative studies have demonstrated that both muscle and fasciocutaneous free flaps yield equivalent overall limb salvage rates and functional outcomes. For instance, a multicenter analysis revealed that although the flap-specific complication profiles varied—with fasciocutaneous flaps being more frequently re-elevated for subsequent orthopedic procedures, the ultimate rates of flap survival, bone union, and long-term functional recovery were similar between the two groups [22,23]. This evidence supports the notion that flap selection should be tailored to the defect’s specific characteristics rather than based solely on historical biases. Recent systematic reviews focusing on older patients with open lower limb fractures have shown comparable complication rates—including flap failure, infection, and reoperations—between free fasciocutaneous and musculocutaneous flaps, underscoring the importance of individualized flap selection based on defect characteristics [24].

When considering the reconstructive strategy, several factors merit attention. Muscle flaps are particularly advantageous in wounds with extensive three-dimensional tissue loss, where their bulk and intrinsic vascularity help fill dead space and protect exposed osseous structures.

However, they often require additional procedures for contouring and may be associated with increased donor-site morbidity. In contrast, fasciocutaneous flaps offer improved esthetic outcomes and facilitate easier subsequent interventions, although their technical demands, especially during perforator dissection, can result in an initially higher complication rate among less experienced surgeons [20,25]. Improving outcomes also depends on surgical team readiness and early recognition of complications. A recent systematic review highlighted that, following complete flap failure, secondary free flap transfer is successful in approximately 69% of cases, while partial failures are more often managed with split-thickness skin grafts (50%) [26].

Ultimately, the decision-making process for lower extremity reconstruction should be multidisciplinary and individualized. Among the many reconstructive options, the profunda artery perforator (PAP) flap has emerged as a valuable alternative in distal lower limb reconstruction, offering favorable outcomes and minimal donor-site morbidity [27].

In the present case, the latissimus dorsi muscle flap was chosen over fasciocutaneous alternatives such as the anterolateral thigh (ALT) or profunda artery perforator (PAP) flaps, based on the circumferential nature of the defect, the need for robust volume to fill dead space, and the anatomical reach required in the distal third of the leg. While ALT flaps offer excellent esthetic outcomes and lower donor-site morbidity, their use was considered suboptimal due to limitations in flap bulk and pedicle orientation in this specific anatomical context.

Additionally, artificial dermal substitutes were also taken into account; however, they were not deemed suitable given the size of the exposed bone area and the need for rapid, vascularized tissue coverage to prevent infection and support healing. Cross-leg flaps, although technically feasible, were excluded due to the expected immobilization time and functional limitations in a young, active patient.

This tailored decision-making process reinforces the importance of adapting flap selection to the defect’s characteristics, patient profile, and long-term functional objectives.

Surgeons must weigh the benefits of a robust, vascularized muscle flap against the superior cosmetic and functional attributes of a fasciocutaneous flap, taking into account factors such as defect size, location, contamination, patient comorbidities, and the anticipated need for secondary procedures. By integrating these considerations, the reconstructive approach can be optimized to achieve both reliable limb salvage and an improved quality of life for the patient.

The versatility of free tissue transfer is exemplified in pediatric cases. Trignano et al. reported a successful heel reconstruction in a 4-year-old using an anterolateral thigh free flap after a severe lawnmower injury, with excellent functional recovery and minimal donor-site morbidity [28]. This case demonstrates that, even in challenging pediatric settings, microsurgical reconstruction can achieve favorable long-term outcomes and supports tailored management of lower limb trauma.

Long-term outcomes following microvascular reconstruction for lower extremity trauma are critical for assessing the overall success of limb salvage procedures. A recent 10-year single-center experience demonstrated that free muscle flaps, while effective in providing durable soft tissue coverage, are associated with substantial early complication rates. In this series, revision surgery was required in approximately 36% of cases and total flap loss occurred in 10.5% of reconstructions. Key predictors for these complications included older age, active smoking, higher ASA scores, and female gender, underscoring the importance of meticulous patient selection and preoperative optimization in minimizing adverse events [29].

In terms of long-term outcomes, a study evaluating patients after successful free flap salvage reported that despite the need for urgent re-explorations and occasional secondary revisions, patients achieved satisfactory functional results. Objective assessments using the Vancouver Scar Scale indicated acceptable scar quality, while patient-reported outcomes via the SF-36 demonstrated good physical and mental health components. These findings suggest that even when early complications occur, prompt and effective intervention can preserve long-term limb function and overall quality of life [30]. Continuous tissue oxygen tension monitoring has also been proposed to enhance early detection of vascular compromise and improve flap salvage [31].

Complementing these results, a multicenter analysis investigating predictors of complications in lower extremity free tissue transfer identified posterior tibial artery injury and immunocompromised status as significant risk factors for both flap failure and major complications. Conversely, achieving flap coverage within 30 days of injury appeared protective. These data highlight that timely reconstruction and thorough vascular assessment are essential to improve outcomes in this challenging clinical scenario [32].

Collectively, these studies indicate that while microvascular free flap reconstruction in lower extremity trauma carries a considerable risk for early complications, long-term outcomes can be favorable with appropriate patient management. Addressing modifiable risk factors such as smoking and ensuring timely intervention are crucial steps toward optimizing functional recovery and enhancing overall patient satisfaction.

Despite the favorable outcome observed in this case, it is important to acknowledge that the findings derived from a single patient experience and cannot be generalized to broader clinical populations.

Additionally, while the 18-month follow-up provides valuable insight into mid-term functional recovery and flap integration, longer follow-up is necessary to fully assess potential late complications, long-term flap durability, and esthetic outcomes.

Negative pressure wound therapy (NPWT) has emerged as a valuable adjunct in managing high-risk lower extremity fractures. A multicenter trial showed that NPWT significantly reduced wound dehiscence and infection rates compared to standard dressings [33]. NPWT also optimizes the wound bed—reducing edema and promoting granulation tissue—which facilitates delayed microsurgical free flap reconstruction [34]. A case report demonstrated that seven days of NPWT before flap coverage in the foot decreased soft tissue defect volume and improved outcomes [13]. These studies collectively underscore NPWT’s ability to lower infection risk and enhance reconstructive success. However, NPWT increases costs and requires specialized equipment and inpatient monitoring. Thus, its use must be judiciously balanced against these limitations. Careful patient selection remains crucial for optimal outcomes in lower extremity reconstruction.

While the use of the latissimus dorsi free flap in lower limb reconstruction is well established, the novelty of this case lies in the rare circumferential configuration of the soft tissue defect, which required a high-volume flap to achieve complete coverage in all compartments of the distal leg. Moreover, the integration of negative pressure wound therapy (NPWT) during the preparatory phase and the demonstration of excellent mid-term functional recovery (LEFS 76/80 at 18 months) further enhance the educational and clinical relevance of this case. To our knowledge, reports of successful microsurgical reconstruction of circumferential lower limb defects using a single muscle flap remain limited in the literature.

## 4. Conclusions

Reconstruction of extensive lower extremity defects following high-energy trauma remains a formidable challenge. Prompt and adequate tissue coverage is essential for successful limb salvage. This clinical case demonstrates that microsurgical free flap reconstruction, particularly with the latissimus dorsi flap, reliably restores structural integrity and function. Adjunctive NPWT optimizes the wound bed and reduces infection risk, enhancing overall outcomes. Timely intervention and careful patient selection are critical in minimizing early complications. Long-term follow-up confirms excellent functional recovery and high patient satisfaction. These results support the latissimus dorsi free flap as the gold standard for managing complex lower limb injuries. Further prospective studies are warranted to refine patient selection and optimize surgical timing.

## Data Availability

The data used or analyzed during the current study are available from the corresponding author upon reasonable request.

## References

[1] Shokrollahi K., Whitaker I.S., Nahai F. (2017). Flaps: Practical Reconstructive Surgery.

[2] Pu L.L.Q., Medalie D.A., Rosenblum W.J., Lawrence S.J., Vasconez H.C. (2004). Free Tissue Transfer to a Difficult Wound of the Lower Extremity. Ann. Plast. Surg..

[3] Hemmann P., Friederich M., Körner D., Klopfer T., Bahrs C. (2021). Changing epidemiology of lower extremity fractures in adults over a 15-year period—A National Hospital Discharge Registry study. BMC Musculoskelet. Disord..

[4] Zhang J., Bradshaw F., Hussain I., Karamatzanis I., Duchniewicz M., Krkovic M. (2024). The Epidemiology of Lower Limb Fractures: A Major United Kingdom (UK) Trauma Centre Study. https://www.cureus.com/articles/188876-the-epidemiology-of-lower-limb-fractures-a-major-united-kingdom-uk-trauma-centre-study.

[5] Godina M. (1986). Early Microsurgical Reconstruction of Complex Trauma of the Extremities. Plast. Reconstr. Surg..

[6] Ciudad P., Agko M., Date S., Chang W., Manrique O.J., Huang T.C.T., Torto F.L., Trignano E., Chen H. (2018). The radial forearm free flap as a “vascular bridge” for secondary microsurgical head and neck reconstruction in a vessel-depleted neck. Microsurgery.

[7] Norris B.L., Kellam J.F. (1997). Soft-Tissue Injuries Associated with High-Energy Extremity Trauma: Principles of Management. J. Am. Acad. Orthop. Surg..

[8] Patterson J.T., Nakata H., Becerra J., Duong A. (2022). Traumatic soft tissue defects: A perspective review on reconstruction and communication priorities from the orthopaedic trauma surgeon as a partner in care. Plast. Aesthetic Res..

[9] Lin C.H. (2021). Functional Restoration in Lower Extremity Reconstruction. Clin. Plast. Surg..

[10] Endara M., Ducic I., Attinger C. (2013). Free Tissue Transfer for Limb Salvage in High-Risk Patients: Worth the Risk. Adv. Wound Care..

[11] Serra P.L., Boriani F., Khan U., Atzeni M., Figus A. (2024). Rate of Free Flap Failure and Return to the Operating Room in Lower Limb Reconstruction: A Systematic Review. J. Clin. Med..

[12] Theodorakopoulou E., Mason K.A., Pafitanis G., Ghanem A.M., Myers S., Iwuagwu F.C. (2016). Free-Tissue Transfer for the Reconstruction of War-Related Extremity Injuries: A Systematic Review of Current Practice. Mil. Med..

[13] Vellingiri K., Nagakumar J.S., Hongaiah D. (2020). Negative Pressure Wound Therapy with Flap Reconstruction for Extensive Soft Tissue Loss in the Foot: A Case Report. https://www.cureus.com/articles/38480-negative-pressure-wound-therapy-with-flap-reconstruction-for-extensive-soft-tissue-loss-in-the-foot-a-case-report.

[14] Krijgh D.D., Van Straeten M.M.E., Mureau M.A.M., Luijsterburg A.J.M., Schellekens P.P.A., Maarse W., Coert J.H. (2020). Postoperative care in microvascular free flap reconstruction of the lower extremity: A systematic review. Orthoplastic Surg..

[15] Hodea F.V., Hariga C.S., Bordeanu-Diaconescu E.M., Cretu A., Dumitru C.S., Ratoiu V.A., Lascar I., Grosu-Bularda A. (2024). Assessing Donor Site Morbidity and Impact on Quality of Life in Free Flap Microsurgery: An Overview. Life.

[16] Dingemans S.A., Kleipool S.C., Mulders M.A.M., Winkelhagen J., Schep N.W.L., Goslings J.C., Schepers T. (2017). Normative data for the lower extremity functional scale (LEFS). Acta Orthop..

[17] Paro J., Chiou G., Sen S.K. (2016). Comparing Muscle and Fasciocutaneous Free Flaps in Lower Extremity Reconstruction—Does It Matter?. Ann. Plast. Surg..

[18] Calderon W., Chang N., Mathes S.J. (1986). Comparison of the Effect of Bacterial Inoculation in Musculocutaneous and Fasciocutaneous Flaps. Plast. Reconstr. Surg..

[19] Ziani F., Rubino C., Manconi A., Arrica G., Trignano C., Ginatempo I., Tettamanzi M., Trignano E. (2025). Complex Post-Traumatic Reconstruction of the Lower Limb: A Case Report on Managing Soft Tissue Defects and Deltoid Ligament Damage Using an ALT Flap With Fascia Lata Extension and Fascia Lata Graft. Ann. Ital. Chir..

[20] Demirtas Y., Kelahmetoglu O., Cifci M., Tayfur V., Demir A., Guneren E. (2010). Comparison of free anterolateral thigh flaps and free muscle-musculocutaneous flaps in soft tissue reconstruction of lower extremity. Microsurgery.

[21] Philandrianos C., Moullot P., Gay A.M., Bertrand B., Legré R., Kerfant N., Casanova D. (2018). Soft Tissue Coverage in Distal Lower Extremity Open Fractures: Comparison of Free Anterolateral Thigh and Free Latissimus Dorsi Flaps. J. Reconstr. Microsurg..

[22] Rodriguez E.D., Bluebond-Langner R., Copeland C., Grim T.N., Singh N.K., Scalea T. (2009). Functional Outcomes of Posttraumatic Lower Limb Salvage: A Pilot Study of Anterolateral Thigh Perforator Flaps Versus Muscle Flaps. J. Trauma-Inj. Infect. Crit. Care.

[23] Cho E.H., Shammas R.L., Carney M.J., Weissler J.M., Bauder A.R., Glener A.D., Kovach S.J., Hollenbeck S.T., Levin L.S. (2018). Muscle versus Fasciocutaneous Free Flaps in Lower Extremity Traumatic Reconstruction: A Multicenter Outcomes Analysis. Plast. Reconstr. Surg..

[24] Kaur A., Ang K.L., Ali S., Dobbs T., Pope-Jones S., Harry L., Whitaker I., Emam A., Marsden N. (2023). Free flaps for lower limb soft tissue reconstruction—A systematic review of complications in ‘Silver Trauma’ patients. Injury.

[25] Yazar S., Lin C.H., Lin Y.T., Ulusal A.E., Wei F.C. (2006). Outcome Comparison between Free Muscle and Free Fasciocutaneous Flaps for Reconstruction of Distal Third and Ankle Traumatic Open Tibial Fractures. Plast. Reconstr. Surg..

[26] Koster I.T.S., Borgdorff M.P., Jamaludin F.S., De Jong T., Botman M., Driessen C. (2023). Strategies Following Free Flap Failure in Lower Extremity Trauma: A Systematic Review. JPRAS Open.

[27] Ciudad P., Kaciulyte J., Torto F.L., Vargas M.I., Bustamante A., Chen H., Maruccia M., Zulueta J., Trignano E., Bolletta A. (2022). The profunda artery perforator free flap for lower extremity reconstruction. Microsurgery.

[28] Trignano E., Serra P.L., Grieco F., Rodio M., Rampazzo S., Pili N., Trignano C., Rubino C. (2023). Heel reconstruction with ALT free flap in a 4-year-old patient after a severe lawnmower injury. A case report. Case Rep. Plast. Surg. Hand Surg..

[29] Besmens I.S., Frueh F.S., Gehrke C., Knipper S., Giovanoli P., Calcagni M. (2023). 10-Year single center experience in lower limb reconstruction with free muscle flaps—Factors influencing complications in 266 consecutive cases. J. Plast. Surg. Hand Surg..

[30] Bigdeli A., Gazyakan E., Schmidt V., Bauer C., Germann G., Radu C., Kneser U., Hirche C. (2019). Long-Term Outcome after Successful Lower Extremity Free Flap Salvage. J. Reconstr. Microsurg..

[31] Trignano E., Fallico N., Fiorot L., Bolletta A., Maffei M., Ciudad P., Maruccia M., Chen H., Gian Vittorio Campus G.V. (2018). Flap monitoring with continuous oxygen partial tension measurement in breast reconstructive surgery: A preliminary report. Microsurgery.

[32] Othman S., Stranix J.T., Piwnica-Worms W., Bauder A., Azoury S.C., Elfanagely O., Klifto K.M., Levin L.S., Kovach S.J. (2023). Microvascular free tissue transfer for reconstruction of complex lower extremity trauma: Predictors of complications and flap failure. Microsurgery.

[33] Stannard J.P., Volgas D.A., McGwin G., Stewart R.L., Obremskey W., Moore T., O Anglen J. (2012). Incisional Negative Pressure Wound Therapy After High-Risk Lower Extremity Fractures. J. Orthop. Trauma..

[34] Raju A., Ooi A., Ong Y., Tan B. (2014). Traumatic Lower Limb Injury and Microsurgical Free Flap Reconstruction with the Use of Negative Pressure Wound Therapy: Is Timing Crucial?. J. Reconstr. Microsurg..

